# Editorial: Targeting neuron-non-neuronal interactions at the neurovascular unit in stroke and neurodegenerative disease models

**DOI:** 10.3389/fncel.2024.1353281

**Published:** 2024-01-26

**Authors:** Changxiang Li, Yang Liu, Jian Liu, Xiaoyu Xu

**Affiliations:** ^1^College of Traditional Chinese Medicine, Beijing University of Chinese Medicine, Beijing, China; ^2^Department of Biochemistry and Molecular Biology, Bloomberg School of Public Health, Johns Hopkins University, Baltimore, MA, United States; ^3^Department of Neuroscience, School of Medicine, Johns Hopkins University, Baltimore, MA, United States; ^4^Peking University Health Science Center, Beijing, China; ^5^College of Traditional Chinese Medicine, Southwest University, Chongqing, China

**Keywords:** stroke, neurodegenerative disease, neuron-non-neuronal cells interactions, neurovascular unit, treatment, mechanisms

## Introduction

Stroke and neurodegenerative diseases continue to be major global contributors to mortality and disability (Pak et al., 2023; Walker et al., 2023). Regrettably, current treatment fall short in addressing post-stroke and neurodegenerative diseases in relation to nervous system recovery. Over the past decades, a wealth of studies (Chang et al., 2016; AlRuwaili et al., 2023; Siddiqui and Bhatt, 2023) have highlighted the potential of neuroprotective and neurorestorative therapies to mitigate brain damage after stroke and neurodegenerative diseases by promoting structural and functional recovery. Most of these therapeutic agents target a single event in the injury cascade and a single neural cell type (Ricciarelli and Fedele, 2017). However, experimental strategies that focus exclusively on vascular or neural targets have not yielded effective therapies in clinics settings. The concept of neurovascular unit (NVU) as a comprehensive target for stroke and neurodegenerative disease treatment has therefore gained traction (Alvarez et al., 2013). The NVU, with its emphasis on multicellular and cell-to-cell interactions, has emerged as a key target in cerebrovascular disease treatment (Wang et al., 2021). Stroke and neurodegenerative diseases can destroy NVU, causing both the structural and functional damage (Alvarez et al., 2023). Non-neuronal cells such as glial cells (astrocytes, microglia, and oligodendrocyte), infiltrating and resident immune cells, and vascular system components (endothelial cells, and pericytes) also play a crucial role in neuronal recovery, with complex signaling cascades that demand further investigation (Lénárt et al., 2023; Ragupathy et al., 2023).

Despite these advances, our understanding of how those cells interact during the healing process, and the mechanisms these interactions trigger that may be beneficial or detrimental to neuroprotection and nervous system regeneration, remains limited. Thus, it is imperative to approach stroke and neurodegenerative diseases with novel mechanistic studies and multifaceted, multi-target neuroprotective and neurorestorative strategies.

The aim of this Research Topic was to explore the treatment and innovative mechanisms of stroke and neurodegenerative diseases, focusing on the interactions between neurons and non-neuronal cells (astrocytes, microglia, oligodendrocytes, pericytes, endothelial cells, monocyte/macrophages, T cells, NK cells, etc.). A total of five papers were included in our Research Topic, including three original articles and two reviews.

## Neurovascular unit

Stroke and neurodegenerative diseases can cause structural and functional damage to the NVU (Li et al., 2020). Each cell type within the NVU plays a vital role, either in the transmission and processing of neural signals or in maintaining the microenvironment necessary for healthy neural function (Li et al., 2019). Therefore, a comprehensive characterization of NVU structure and function is crucial to understanding the pathology of stroke and neurodegenerative diseases. *In vitro* NVU culture models recapitulate brain-specific functions and offer greater experimental control over the cellular and molecular interactions under investigation than *in vivo* models (Uwamori et al., 2017). Moreover, the exploration of multi-targeted brain cytoprotective agents has emerged as a crucial direction for future treatment of stroke and neurodegenerative diseases.

In this Research Topic, a review by Novorolsky et al. potentially illuminates a promising therapeutic avenue for ischemic or hemorrhagic stroke. Their previous investigations have validated the roles of the mitochondrial Ca^2+^ uniporter complex (MCUcx) and the sodium/Ca^2+^/lithium exchanger (NCLX) in mediating the uptake and efflux of mitochondrial Ca^2+^ in the brain. The review highlights the potential of nanoparticle-based approaches to enhance clinical safety and efficacy by optimizing drug delivery to diseased NVUs and limiting drug exposure in healthy brain and peripheral tissues. Furthermore, in a *vitro* experiment, Zhang et al. established induced pluripotent stem cell (iPSC) models from Cerebral Autosomal Dominant Arteriopathy with Subcortical Infarcts and Leukoencephalopathy (CADASIL) patients and evaluated the blood brain barrier (BBB) function by measuring transendothelial electrical resistance (TEER). Their study demonstrates the importance of neurovascular interaction and BBB function at the molecular and cellular levels for CADASIL.

## Neuron-non-neuronal interractions

Stroke and neurodegenerative diseases may lead to the destruction of all brain cell types, disrupting the relative balance among them (Shabir et al., 2018; Le Roy et al., 2021). Unfortunately, a large proportion of studies aiming to develop neuroprotective agents only target neurons, neglecting their interactions with other brain cells types and thus failing to reflect the *in vivo* brain characteristics (Li et al., 2021). Non-neuronal cells, including glial cells (astrocytes, microglia, and oligodendrocyte), infiltrating and resident immune cells, and the vascular system (endothelial cells, and pericytes), possess critical specific signals and execution cascades that can promote neuronal recovery (Venkat et al., 2018). Consequently, the development of neuroprotective agents should aim to protect a variety of cell types and coordinate their interactions, rather than focusing solely on neurons.

In this Research Topic, Charlton et al. suggest that brain-derived neurotrophic factor (BDNF) exerts direct anti-inflammatory effects on microglia. They discovered that BDNF treatment significantly reversed the release of both IL-6 and TNF- in primary cortical microglia following LPS-induced inflammation. This led to the hypothesis that BDNF may directly modulate microglia state, thereby influencing microglia-neuron interactions. Yuan et al. provided a comprehensive overview of the ceramide synthesis pathways and highlighted their dysregulation in stroke, cerebral small vessel disease (CSVD), and related risk factors, focusing on the underlying mechanism in different types of stroke and CSVD. They demonstrated that NVU exhibits intricate contact in a coordinated manner to control the blood-brain barrier (BBB), regulate cerebral perfusion, and maintain microenvironment homeostasis. When ischemia and hypoxia were stimulated, astrocytes were induced by two different phenotypes known as A1 and A2. Feng et al. demonstrated that 2,3,5,6-Tetramethylpyrazine (TMP) can treat ischemic stroke via the FGF2/PI3K/AKT pathway, with the astrocyte transformation into the anti-inflammatory A2 subtype playing an important role in NVU preservation and neurovascular remodeling after ischemic stroke.

## Conclusion

This Research Topic encompasses five articles that cover a broad range of neuron-non-neuronal interactions at the NVU in stroke and neurodegenerative diseases. Several potential therapeutic targets for these conditions were identified, including BDNF, MCUcx, NCLX, and ceramide. TMP was highlighted as a promising candidate for the treatment of ischemic stroke. A more complex *in vitro* NVU system was established to better mimic the cerebral vasculature and to explore pathological mechanisms. These discoveries significantly advance our understanding of the potential molecular mechanisms and therapeutic effects in stroke and neurodegenerative diseases.

## Author contributions

CL: Writing – original draft, Writing – review & editing. YL: Writing – original draft, Writing – review & editing. JL: Writing – original draft, Writing – review & editing. XX: Writing – original draft, Writing – review & editing.
